# Prospective evaluation of the diagnostic value of plasma apelin 12 levels for differentiating patients with moyamoya and intracranial atherosclerotic diseases

**DOI:** 10.1038/s41598-017-05664-8

**Published:** 2017-07-14

**Authors:** Wei Hu, Wan Jiang, Li Ye, Yanghua Tian, Bing Shen, Kai Wang

**Affiliations:** 1 Department of Neurology, Affiliated Provincial Hospital of Anhui Medical University, Hefei, Anhui 230032 China; 2 Department of Neurology, The First Affiliated Hospital of Anhui Medical University, Hefei, Anhui 230032 China; 3 School of Basic Medical Sciences, Anhui Medical University, Hefei, Anhui 230032 China

## Abstract

Patients with moyamoya disease (MMD) or intracranial atherosclerotic disease (ICAD) experience similar cerebral ischaemic events. However, MMD patients show greater angiogenesis and arteriogenesis, which play crucial roles in collateral circulation development to enhance clinical prognosis and outcome. Apelins have been associated with angiogenesis and arteriogenesis. Therefore, the aim of the present study was to determine whether apelin levels were higher in patients with MMD than in patients with ICAD or in healthy controls. We compared plasma apelin levels in 29 patients with MMD, 82 patients with ICAD, and 25 healthy participants. Twelve-hour fasting blood samples were collected and analysed using commercially available kits. Univariate analyses indicated that compared with the ICAD and healthy control groups, the MMD group had higher apelin-12, apelin-13, apelin-36, and nitric oxide levels. Binary logistic regression analyses further showed that the plasma apelin-12 level was substantially higher in MMD patients than in ICAD patients. Patients with MMD were also differentiated from patients with ICAD by their mean ages, with the former being younger. Therefore, the plasma apelin-12 level is a potential diagnostic marker for differentiating MMD and ICAD and may provide a treatment strategy for enhancing collateral circulation development and clinical prognosis and outcome.

## Introduction

Moyamoya disease (MMD) is an idiopathic disease characterized by spontaneous stenosis and occlusions of distal internal carotid arteries and their major branches^1^. Although the prevalence of MMD is higher in East Asian countries, including Japan, Korea and China^2, 3^, MMD has been observed in various populations worldwide^4^. The frequency of transient ischaemic attacks in patients with MMD and chronic cerebral ischaemia is similar to that in patients with other aetiologies. However, compared with patients diagnosed with chronic cerebral ischaemia, patients with MDD have greater capacities for arteriogenesis and angiogenesis as well as better clinical prognoses and outcomes^5^. Many studies have shown that apelin is closely related to angiogenesis^6–8^ and promotes angiogenesis after ischaemic stroke^9^.

Apelin, an endogenous peptide that was first isolated from bovine stomach tissue in 1998, binds to its G protein-coupled receptor APJ^10^. Apelin is expressed in the human gastrointestinal tract, heart, brain, kidney, liver, adipose tissue, endothelium and plasma^11^. Apelin has been reported to decrease vascular tone through a nitric oxide (NO)-dependent mechanism^12^. It is known that NO plays a crucial role in regulating the basal tone of cerebral parenchymal arterioles and increasing collateral circulation in cerebral vascular occlusive disease, such as MMD^13^. However, many studies have also indicated that apelin may increase the expression of vascular endothelial growth factor (VEGF) to enhance angiogenesis^14–21^. Moreover, several additional reports have shown that apelin may cooperate with VEGF to enhance angiogenesis and may be involved in the treatment of stroke and MMD^22–27^.

Although MMD and intracranial atherosclerotic disease (ICAD) are both classified as intracranial cerebral artery diseases, patients with MDD have greater capacities for arteriogenesis and angiogenesis^5^. Therefore, we hypothesised that patients with MMD may have higher levels of plasma apelin and NO than patients with ICAD and healthy persons, and thus aimed to determine whether plasma apelin or NO levels could be used as markers to differentiate patients with MMD from those with ICAD.

## Results

**Figure 1:**
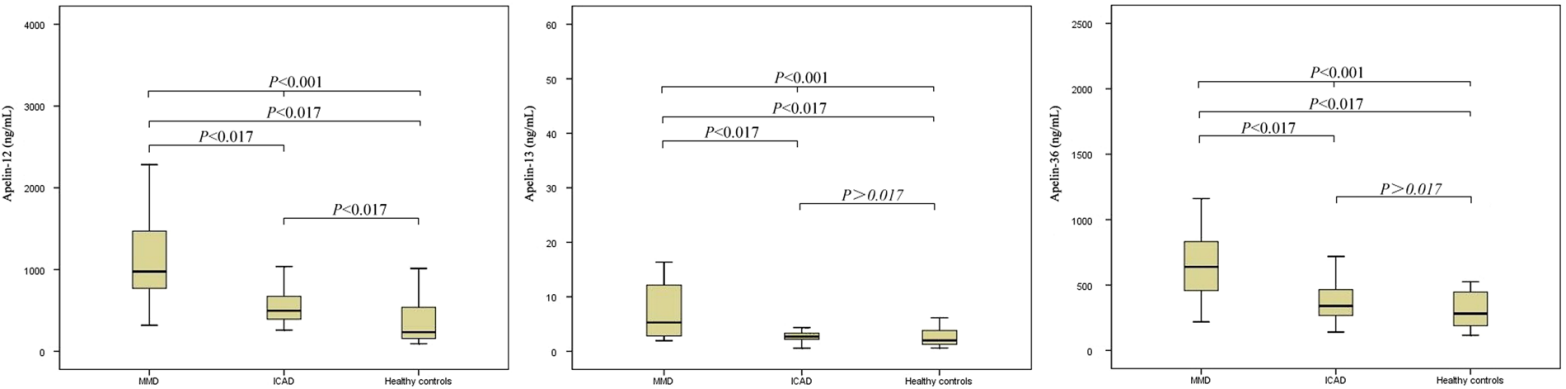
Apelin-12, apelin-13, and apelin-36 levels in patients with moyamoya disease (MMD) or intracranial atherosclerotic disease (ICAD) and in healthy controls.

A total of 29 patients with MMD, 82 patients with ICAD, and 25 healthy participants were included in this study. The characteristics of the participants are shown in Table 1. The mean age, ratio of males to females, and systolic blood pressure (SBP) were significantly higher in the ICAD group than in the other two groups. Levels of apelin-12, apelin-13, apelin-36, and NO were significantly higher in the MMD group than in the other two groups (Fig. 1 and Table 1). Plasma apelin-12 and NO levels were significantly higher in patients with ICAD than in healthy participants (Table 1).

**Table 1:** Univariate analysis for Control, MMD, and ICAD groups.

Parameter	Control group	MMD group	ICAD group	*P* value
Age, years^a^	49.08 ± 9.36	51.21 ± 12.02	62.11 ± 12.08^d^	<0.001
BMI (kg/m^2^)^a^	23.63 ± 2.57	23.65 ± 2.65	24.62 ± 3.09	0.162
SBP (mmHg)^a^	131.40 ± 5.63	131.00 ± 17.35	145.13 ± 19.74^d^	<0.001
DBP (mmHg)^a^	81.04 ± 5.59	79.48 ± 10.18	83.54 ± 12.28	0.196
Total cholesterol (mg/dl)^a^	4.24 ± 0.46	4.23 ± 1.22	4.21 ± 1.00	0.987
LDL-cholesterol (mg/dl)^a^	2.60 ± 0.55	2.36 ± 0.94	2.58 ± 0.83	0.409
HDL-cholesterol (mg/dl)^a^	1.26 ± 0.13	1.25 ± 0.36	1.18 ± 0.39	0.438
Lipoprotein a (mmol/l)^a^	196.52 ± 76.44	227.93 ± 251.37	274.74 ± 233.78	0.244
Blood glucose (mmol/l)^b^	4.90 ± 0.75	4.90 ± 1.34	5.20 ± 1.57	0.199
Triglyceride (mg/dl)^b^	1.50 ± 0.38	1.81 ± 0.99	1.48 ± 0.90	0.476
Homocysteine (nmol/l)^b^	11.05 ± 3.24	14.80 ± 5.95	13.58 ± 6.27	0.571
Apelin-12 (ng/dl)^b^	233.50 ± 405.21	975.44 ± 755.39^e^	496.41 ± 284.72^d^	<0.001
Apelin-13 (ng/dl)^b^	2.00 ± 3.26	5.28 ± 9.66^e^	2.70 ± 1.15	<0.001
Apelin-36 (ng/dl)^b^	282.05 ± 266.93	639.04 ± 488.77^e^	339.95 ± 201.36	<0.001
NO (nmol/l)^b^	23.26 ± 48.23	59.01 ± 70.89^e^	36.02 ± 29.25^d^	<0.001
Sex, male (%)^c^	48.00	41.38	68.29^d^	0.019
Smoke cigarettes (%)^c^	16.00	24.14	35.37	0.138
Consume alcohol (%)^c^	16.00	10.34	21.95	0.361

^a^Data are presented as mean ± SD; ^b^Data are presented as median ± IQR; ^c^Data are presented as %. BMI, body mass index; SBP, systolic blood pressure; DBP, diastolic blood pressure; NO, Nitric oxide; MMD, moyamoya disease; ICAD, intracranial atherosclerotic disease. ^d^Significant difference vs. Control group and MMD group (*P* < 0.017). ^e^Significant difference vs. Control group and ICAD group (*P* < 0.017).

Multivariate logistic regression analyses were used to investigate significant factors differentiating the MMD, ICAD, and healthy participant groups. We found that the mean age, apelin-12 level, and SBP were significantly higher in the MMD and ICAD groups than in the healthy participant group (Table 2).

**Table 2:** Multivariate logistic regression analysis for Control, MMD, and ICAD groups.

Parameter		B	S.E.	Wald	P value	Odds ratio (95% CI)
MMD group versus Control group	Intercept	−14.457	6.921	4.364	0.037	
	Age	0.009	0.038	0.057	0.811	1.009 (0.937–1.086)
	Apelin-12	0.011	0.002	20.915	<0.001	1.011 (1.006–1.015)
	Apelin-13	0.164	0.161	1.037	0.308	1.178 (0.859–1.616)
	Apelin-36	−0.002	0.001	1.349	0.246	0.998 (0.995–1.001)
	NO	−0.013	0.017	0.586	0.444	0.987 (0.954–1.021)
	SBP	0.069	0.032	4.733	0.030	1.071 (1.007–1.140)
ICAD group versus Control group	Intercept	−24.317	6.248	15.146	<0.001	
	Age	0.117	0.032	13.412	<0.001	1.124 (1.056–1.197)
	Apelin-12	0.008	0.002	13.306	<0.001	1.008 (1.004–1.012)
	Apelin-13	0.024	0.151	0.025	0.873	1.024 (0.761–1.378)
	Apelin-36	−0.001	0.001	0.796	0.372	0.999 (0.997–1.001)
	NO	−0.020	0.014	1.946	0.163	0.981 (0.954–1.008)
	SBP	0.075	0.026	8.186	0.004	1.077 (1.024–1.134)

Regression model was adjusted by BMI, sex, cigarette smoking, and alcohol consumption. BMI, body mass index; SBP, systolic blood pressure; NO, Nitric oxide; MMD, moyamoya disease; ICAD, intracranial atherosclerotic disease.

Binary logistic regression analyses indicated that the plasma apelin-12 level in the MMD group was significantly higher than that in the ICAD group, and the mean age of the patients in the MMD group was significantly lower than that in the ICAD group (Table 3, model 1). Binary logistic regression was also used to analyse each of the three apelins and NO independently. The results demonstrated that apelin-13, apelin-36, and NO were significantly increased in patients with MMD compared with those in the ICAD group, and the mean age of the patients in the MMD group was significantly lower than that of the ICAD group (Table 3, models 2–5).

**Table 3:** Binary logistic regression analysis for MMD and ICAD groups.

Model	Parameter	B	S.E.	Wald	*P* value	Odds ratio (95% CI)
1	Age	0.099	0.030	11.128	0.001	1.104 (1.042–1.170)
	Apelin-12	−0.002	0.001	6.274	0.012	0.998 (0.996–0.999)
	Apelin-13	−0.145	0.116	1.578	0.209	0.865 (0.689–1.085)
	Apelin-36	0.001	0.001	0.467	0.494	1.001 (0.999–1.003)
	NO	−0.007	0.014	0.241	0.624	0.993 (0.966–1.021)
	SBP	0.006	0.020	0.080	0.778	1.006 (0.967–1.046)
	Constant	−11.893	4.938	5.800	0.016	0.000
2	Age	0.092	0.027	11.525	0.001	1.097 (1.040–1.156)
	Apelin-12	−0.003	0.001	15.672	<0.001	0.997 (0.995–0.998)
	SBP	0.012	0.019	0.410	0.522	1.012 (0.975–1.050)
	Constant	−13.090	5.011	6.824	0.009	0.000
3	Age	0.079	0.025	9.877	0.002	1.082 (1.030–1.136)
	Apelin-13	−0.282	0.088	10.257	0.001	0.754 (0.634–0.896)
	SBP	0.020	0.017	1.345	0.246	1.020 (0.986–1.055)
	Constant	−11.176	4.347	6.610	0.010	0.000
4	Age	0.064	0.024	7.360	0.007	1.066 (1.018–1.117)
	Apelin-36	−0.002	0.001	5.687	0.017	0.998 (0.997–0.999)
	SBP	0.030	0.016	3.556	0.059	1.030 (0.999–1.062)
	Constant	−11.509	4.206	7.487	0.006	0.000
5	Age	0.080	0.025	10.115	0.001	1.083 (1.031–1.137)
	NO	−0.031	0.010	8.900	0.003	0.970 (0.950–0.990)
	SBP	0.019	0.017	1.222	0.269	1.019 (0.986–1.053)
	Constant	−9.981	4.243	5.534	0.019	0.000

All regression models were adjusted by BMI, sex, cigarette smoking, and alcohol consumption. BMI, body mass index; SBP, systolic blood pressure; NO, Nitric oxide; MMD, moyamoya disease; ICAD, intracranial atherosclerotic disease.

**Figure 2:**
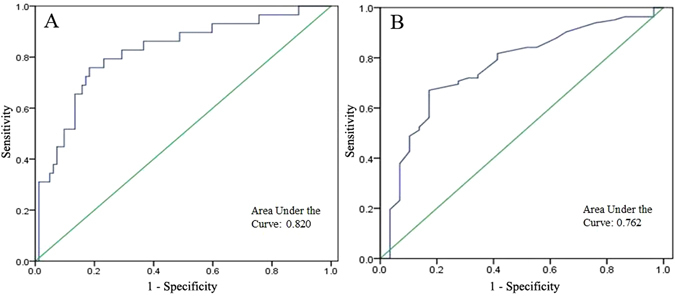
Receiver operating characteristic (ROC) curve analyses of apelin-12 (**A**) and age (**B**) as predictors of moyamoya disease (MMD).

Receiver operating characteristic (ROC) curve analyses indicated that in differentiating MMD from ICAD, an apelin-12 level of 759.97 ng/mL had a 76% sensitivity and an 82% specificity (Fig. 2A) and 57.5 years old had a 67% sensitivity and an 83% specificity (Fig. 2B). Using Pearson’s correlation analyses that included all participating patients, we found significant correlations between plasma apelin levels and serum NO concentrations or age (Table 4).

**Table 4:** Pearson’s correlation results between serum nitric oxide (NO) and plasma apelin levels in all participating patients (both MMD and ICAD groups).

Parameter	NO		Age	
	r	*P* value	r	*P* value
Apelin-12	0.626	<0.001	−0.098	0.401
Apelin-13	0.822	<0.001	−0.143	0.134
Apelin-36	0.586	<0.001	−0.206	0.030

## Discussion

In the present study, we found significantly increased plasma apelin-12 levels in patients with MMD compared with those in patients with ICAD and in healthy persons. Age was shown to be a significant factor differentiating MMD from ICAD, as patients with MMD were significantly younger than those with ICAD.

Apelin is produced as an immature 77-amino acid prepropeptide that can be cleaved by proteases into C-terminal fragments, including apelin-12, apelin-13, and apelin-36^28^. Apelins are widely expressed in neuronal cell bodies and fibres throughout the entire neuraxis^29^. We found that plasma apelin-12 levels were significantly higher in patients with MMD and ICAD than in healthy participants. Moreover, apelin-12, apelin-13 and apelin-36 levels were also significantly higher in patients with MMD than in patients with ICAD. However, in binary logistic regression analyses, neither apelin-13 nor apelin-36 levels were found to be significantly different between the patient groups. Many recent studies have indicated that hypoxia leads to an increase in apelin transcription and expression in tissues^30–32^. In the present study, the patients with MMD or ICAD had an abnormal brain blood supply, which leads to brain ischaemia and hypoxia. Thus, the brain hypoxia in these patients may have caused their CNS neurons and endothelial cells to express and release more apelin. Some MMD patients have a congenital arteriovenous malformation and a long course of disease. By contrast, atherosclerosis usually occurs in patients aged more than 40 years^33^. Therefore, a longer course of MMD together with the severity of hypoxia observed in MMD may explain why the level of apelin-12 was higher in patients with MMD than in patients with ICAD.

One study reported that apelin protects the brain from ischemia/reperfusion injury in a mouse model of focal transient cerebral ischaemia^34^. Intraperitoneal injection of apelin-12 induces the expression of c-Fos protein in several brain nuclei to enhance the transcription of interleukin-6 and vascular endothelial growth factor α, which promotes angiogenesis^32^. By contrast, lower apelin levels have a negative effect on vascular sprouting and angiogenesis^22^. Moreover, gene therapy that combines VEGF and apelin has been reported as a potential option for treating patients with MMD^35^. Therefore, the higher apelin levels in patients with MMD may explain their greater capacity for arteriogenesis and angiogenesis and their better clinical prognoses and outcomes compared with those for patients with ICAD^5^, as well as provide a better understanding of the pathology and aetiology of MMD. Importantly, our findings also suggest that apelin-12 may be a candidate useful for the development of drugs to treat brain ischaemic disease. In addition, our ROC curve analyses showed that apelin-12 levels may have diagnostic value for differentiating MMD and ICAD.

Recently, Azizi and colleagues found that apelin enhances NO production^36^. Our data also revealed that plasma NO levels were significantly increased in MMD and ICAD patients and that there was a significant correlation between NO concentrations and apelin levels. However, another study has determined that the nitrate and nitrite levels in patients with MMD are not different from those in healthy participants and that NO metabolite levels are decreased in patients with intracranial atherosclerotic stroke^37^. Therefore, further studies will be needed to confirm the NO changes observed in the patients with MMD and ICAD in the present study.

The incidence of MMD in China reportedly peaks in two age groups: in 10-year-olds (approximately) and in 30–40-year-olds^38^. But patients with atherosclerosis are generally older than 40 years of age^33^. Indeed, we found that patients with ICAD were older than those with MMD. However, in order to more closely match the ages of the patients with ICAD in the present study, juvenile patients with MMD were excluded from the present study. An age-dependent decrease in apelin production might be responsible for the lower apelin levels observed in the ICAD group, which showed an inverse correlation between apelin level and age.

Previous studies have shown that hypertension is an independent risk factor for intracranial atherosclerosis^39, 40^. MMD, an idiopathic vascular disorder of intracranial arteries, is often accompanied by hypertension^41^. In the present study, univariate analysis found that systolic blood pressure in patients with ICAD was higher than that in patients with MMD, who were also significantly younger. Because age is a well-known independent risk factor for hypertension, the difference in systolic blood pressure may also be age-related.

In conclusion, our results indicated that the plasma apelin-12 level was significantly increased in patients with MMD and ICAD. Furthermore, the plasma apelin-12 level was substantially higher in patients with MMD than in patients with ICAD. Although the interpretation of our results may be limited by the relatively small sample sizes and the simple experimental design used in this study, the difference we observed in apelin levels between patients with MMD and ICAD suggests that apelin-12 has a potential diagnostic value in differentiating MMD from ICAD. Moreover, apelin-12 may also be a promising candidate for use in developing clinically therapeutic drugs for ischaemic brain disease although further studies confirming our findings are warranted.

## Methods

### Study design

Informed consent to publish identifying information/images was obtained from all participants or their family members for the present study. All study procedures were approved by the Ethics Committee of the Affiliated Provincial Hospital of Anhui Medical University and conducted according to the Helsinki Declaration (1975 and subsequent revisions).

### Participants

The study population included patients who had undergone brain imaging by computed tomography (CT) or magnetic resonance imaging (MRI) to discover the presence of clinical ischaemic or haemorrhagic symptoms in combination with vascular lesions. Patients with MMD were diagnosed by digital subtraction angiography to find the stenosis or occlusion at the terminal portion of the carotid arteries with arterial collateral vessels at the base of brain. For diagnosis of ICAD, computed tomography angiography was used to detect the stenosis and plaque at the intracranial artery. Patients were excluded from enrolment for contraindication to iodinated contrast agent administration (history of contrast agent allergy, pregnancy, congestive heart failure, and renal insufficiency), previous revascularization history (cerebral artery bypass grafting or cerebral artery intervention), evidence of ongoing infection or inflammation or autoimmune diseases, chronic obstructive pulmonary disease, previous diagnosis of malignancy, diabetes with a history of insulin usage, body mass index (BMI) of ≤20 kg/m^2^ or ≥30 kg/m^2^, or a non-enhanced CT scan showing evidence of intracranial haemorrhage.

A total of 29 adult patients with MMD and 82 adult patients with ICAD were admitted to our clinic between 2013 and 2016. In order to more closely age-match the patient groups, juvenile patients with MMD were excluded from the study. Twenty-five healthy volunteers working as staff in our section were recruited for the control group. Blood pressure, BMI, and biochemical parameters were evaluated three times to ensure data accuracy.

### Analysis of lipids, nitric oxide, and apelin

Blood samples were collected after a 12-h fast. Biochemical and lipid parameters were measured using an automatic enzymatic analyser (Beckman Coulter, AU5800, Japan). For apelin, 5-mL blood samples were collected in tubes containing EDTA-K2. Immediately following centrifugation at 3600 rpm for 10 min, the plasma samples were frozen at −80 °C until analyses were conducted. Apelin-12, apelin-13, and apelin-36 levels were assayed using commercially available enzyme immunoassay kits (Phoenix Pharmaceuticals, Burlingame, CA, USA). For NO, blood (5 mL) was collected and centrifuged at 3600 rpm. The serum samples were separated and stored at −80 °C. The serum NO level was determined using a nitrate/nitrite colorimetric assay kit (Cayman Chemical, Michigan, USA).

### Statistical analysis

Continuous variables are presented as the mean ± standard deviation (SD) or median ± interquartile range; categorical variables are presented as percentages. The normal distribution of numeric variables was assessed with the Kolmogorov-Smirnov test. Differences in categorical variables among the groups were examined using the χ^2^ test. Comparisons of continuous variables were analysed with one-way analysis of variance, Mann–Whitney *U*, or Kruskal–Wallis tests. ROC curves were constructed to establish the predictive values of apelin and age for the differentiation of MMD from ICAD, and the optimal cut-off values of apelin and age were used in the binary logistic regression model. Factors were tested in univariate analysis, and those factors with values of *P* < 0.1 were tested in binary logistic regression analysis (Method: enter) and multivariate logistic regression analysis (Method: enter), which was adjusted by BMI, sex, cigarette smoking, and alcohol consumption. Correlations were analysed with Pearson’s correlation. Values of *P* < 0.05 or <0.017 (0.05/3 = 0.017) were considered to be statistically significant. All tests were two-tailed, and all analyses were performed using Statistical Package for Social Sciences software (SPSS, version 17.0; SPSS Inc., Chicago, IL, USA).
